# Propensity Score Matching Analysis of the Effect of Payer Status on the Survival of Colon Cancer Patients

**DOI:** 10.7759/cureus.15748

**Published:** 2021-06-18

**Authors:** Lawrence Shi, Winston Suh, Mindie M Kavanaugh, Glenn Mills, Sarah Thayer, Runhua Shi

**Affiliations:** 1 Hematology-Oncology, Tulane University, New Orleans, USA; 2 Hematology-Oncology, Department of Medicine & Feist-Weiller Cancer Center, Louisiana State University Health Shreveport, Shreveport, USA; 3 Surgical Oncology, Department of Medicine & Feist-Weiller Cancer Center, Louisiana State University Health Shreveport, Shreveport, USA

**Keywords:** payer status, colon cancer, retrospective study, healthcare insurance, propensity score matching (psm)

## Abstract

Background and objective

Colon cancer is one of the most common types of cancer globally. The factors that could affect colon cancer survival include age, stage, treatment, and other socioeconomic aspects. Payer status has been shown to be a significant predictor of cancer patient survival in retrospective studies. However, due to the limitations of retrospective studies, patient baseline characteristics between payer statuses are not comparable. Few studies have addressed the effect of payer status on the overall survival (OS) of patients using propensity score matching (PSM). In light of this, we conducted a study to examine the effect of payer status on the survival of colon cancer patients based on PSM.

Materials and methods

About 66,493 stage II/III colon cancer patients aged 40-90 years and diagnosed between 2004 and 2015 were analyzed from a de-identified National Cancer Database (NCDB) file. All patients had undergone surgery, and patients who had received radiation therapy, hormone therapy, immunotherapy, palliative care, or therapies other than chemotherapy were excluded. Only private or Medicaid payer status was included. The propensity score was calculated by computing the probability of patients being in the Medicaid group using logistic regression. The PSMATCH procedure in the SAS software (SAS Inc., Gary, NC) was used to perform PSM on patients with Medicaid and private insurance. The greedy nearest neighbor matching method was used to match one Medicaid to one privately insured patient with a caliper of 0.2. At the same time, an exact match was done for gender, age group, race, and stage at diagnosis. Multivariate Cox regression was then used to estimate the effect of payer status on survival before and after PSM.

Results

Among the 66,493 patients, 90.3% were privately insured and 9.7% had Medicaid. In univariate analysis, payer status was found to be a significant predictor of OS. Prior to PSM, the median overall survival (MOS) for patients with private insurance was 12.75 years, while those with Medicaid had a MOS of 9.02 years. After PSM, 6,167 paired patients were matched, and patients with private insurance had a MOS of >12.82 years and Medicaid patients had a MOS of 8.88 years. After PSM, patients with Medicaid had a 50% increased risk of death, and payer status proved to be a statistically significant predictor of OS of colon cancer.

Conclusion

Based on our findings, as per the PSM method, payer status can be a significant predictor of survival among colon cancer patients. Also, chemotherapy, race, age, and other socioeconomic factors were also found to be significant predictors of OS. Further research should be conducted to investigate other covariates not studied here and the mediation effect of payer on the survival of cancer patients.

## Introduction

As healthcare reform in the United States continues to evolve, defining the impact of payer status on health outcomes remains a challenging prospect. Several studies have uncovered a link between uninsured/underinsured payer status and higher risks of mortality from cancer [1-8]. Many expect the shift in insurance coverage across the United States to continue [9-13], and the effect that this shift will have on cancer patients is still uncertain.

Colon cancer is a very common disease and accounts for a third of all cancer-related deaths in the United States among both men and women. From 2012-2016, new cases of colorectal cancer for men and women amounted to 38.6 per 100,000 people, and there were 14.2 deaths per 100,000 people per year. Among both genders, blacks had the highest number of new cases and deaths per 100,000 persons. The American Cancer Society estimates that there will be 149,500 new cases of colorectal cancer (104,270 colon and 45,230 rectal cancer cases) and 52,980 deaths in the United States in 2021 [14]. While the Surveillance, Epidemiology, and End Results (SEER) data from 2009-2015 has reported an overall 64.4% five-year relative survival rate with the highest percentage of colorectal cancer deaths among people aged 75-84 [15], colorectal cancer-related deaths have been dropping for several decades due to technological and medical advances that allow for earlier detection and a higher quality of treatment, respectively [14]. As a result, there are now more than 1.5 million colon cancer survivors in the United States, and the number is continuing to rise [14]. Fortunately, the survival rate from colorectal cancer has been steadily increasing over the past 20 years, likely due to greater participation in screening, earlier diagnoses, and improvements in treatment [16,17]. However, as these advances become more widely available to those with insurance, we must also consider the impact this might have on healthcare coverage disparities.

The impact of demographic, socioeconomic, and geographic disparities on the overall survival (OS) of patients with colorectal cancer has been studied extensively [18-28]. The black population has been associated with significantly worse OS compared to other groups, due to factors such as fewer as well as lower quality screenings [19,22] or a greater likelihood to be diagnosed with late-stage cancers [23]. Regarding gender, women have been shown to have better OS compared to men [18]. Diagnoses that are more recent have been associated with better OS (likely due to advances in treatment) while older age has been linked to a greater risk of proximal tumors [18], which have ultimately been shown to have worse mortality rates [29].

Other studies have shown patients from socioeconomically deprived neighborhoods to be at greater risk of death from colorectal cancer [20,21], while a lower education acts as a barrier to proper cancer screening uptake [24]. Patients who live in less urbanized areas have been shown to refuse treatment more often, likely due to lack of access [21], and longer distances to screening and treatment centers have been associated with a greater chance to present with metastatic disease [30] or further obstruction to quality care [31]. Patients who present with comorbidities have been shown to have a higher overall risk of death [20] while chemotherapy treatment has been linked to better survival outcomes [32]. Any significant delay in treatment from the point of diagnosis can be detrimental to prognosis [21]. Even facility type has been shown to be a key factor with treatment at a community care cancer center linked to a greater risk of death [33,34].

The focus of this study is the effect of payer status on colon cancer survival. Some studies on colorectal cancer have linked data between the SEER and Center for Medicare Service to investigate the effect of these variables on survival, but not with payer status. The role of payer status in OS has only recently begun to garner attention, and recent studies have demonstrated a link between the two [6,35-37]. One study has concluded that Medicaid and uninsured patients had a >40% higher risk of death compared to patients with private insurance [5]. Another study has revealed similar findings and shown that Medicaid and uninsured patients had worse OS compared to other patients, even after adjusting for other variables [3].

A statistically significant relationship between payer status and cancer patient survival has been demonstrated in previously published retrospective studies [33,38]. However, in retrospective studies, patient baseline characteristics between payer status are not comparable [32]. If payer status was shown to have an effect on the OS, such an effect could then be due to other unbalanced factors. To overcome the lack of comparability of baseline characteristics in retrospective studies, the propensity score matching (PSM) method can be used to balance patient characteristics between payer status groups [32,39]. Thus, this study has employed the PSM method to assess the effect of payer status on the survival of colon cancer patients.

## Materials and methods

This study examined 66,493 patients with stage II or III colon cancer who were registered between 2004 and 2015 and followed up until December 31, 2015. The data was derived from a de-identified National Cancer Database (NCDB) file. The NCDB documents approximately 70% of all newly diagnosed cases of cancer in the United States at the institutional level [31]. Codes from the International Classification of Disease for Oncology, third edition (ICD-O-3) associated with a diagnosis of colon cancer (C180-189) were used to select patients. All patients had undergone surgery, and patients who had received radiation therapy, hormone therapy, immunotherapy, palliative care, or therapies other than chemotherapy were excluded. Only patients with private insurance or Medicaid were included, while those with unknown insurance, Medicare, and other insurance types were excluded.

The survival duration of colon cancer patients was calculated from the date of diagnosis to the date of death, date of loss to follow-up, or date of study conclusion (December 31, 2015). The variables investigated included payer status, sex, age, race, Charlson Comorbidity score, income, education, distance traveled, facility type, diagnosing/treating facility, treatment delay, stage, year of diagnosis, and chemotherapy.

The patients were classified into two groups based on age: those aged 40-64 years and those of 65+ years. Patient race was categorized as white or black. Charlson Comorbidity [32] is an index that characterizes the overall health status of a patient. Charlson Comorbidity was categorized as “yes” or “no” to indicate whether a patient did or did not present with a comorbidity along with initial cancer diagnosis. Zip Code level income and education were also used as variables in this research. Patients were further classified into different groups based on income (household income of <36,000 or >36,000 USD per year) as well as education (>20% to denote at least a high school education while <20% denoted those who did not receive a high school education). Distance traveled to the facility was defined as <30 or >30 miles and the facility type was determined based on the NCDB’s categorization of Comprehensive Community Cancer and Academic/Research program. Diagnosing/treating facility was categorized based on whether patients were diagnosed and treated at the same initial facility or a different facility. Treatment delay from diagnosis was grouped as 0-30 or >31 days; tumor stage was defined based on the American Joint Committee on Cancer’s (AJCC) categorization of stage II and III tumors; classification based on years of diagnosis included patients diagnosed between 2004-2009 and those between 2010-2014. The treatment status was categorized as either “received” or “not received” for chemotherapy.

The propensity score was calculated by computing the probability of patients being in the Medicaid group using logistic regression. The PSMATCH procedure in the SAS software (SAS Inc., Gary, NC) was used to perform PSM [30] on patients with Medicaid and private insurance. The greedy nearest neighbor matching method [39] was used to match one Medicaid to one privately insured patient with a caliper of 0.2. At the same time, an exact match was done for gender, age group, race, and stage at diagnosis. A caliper measure such as “caliper=0.5” is the caliper requirement used for matching, meaning that the difference in PS logits must be less than or equal to 0.5 times the estimated logit of the standard deviation.

Patient characteristics were compared between private and Medicaid patients before and after matching by using the chi-square test. The Kaplan-Meier/product-limit method was used to estimate the survival of the patients. The log-rank test was used to compare the OS for payer status. Multivariate Cox regression was used to simultaneously estimate the hazard of death [hazard ratio (HR)] for payer status and adjust for other factors. The statistical software SAS 9.4 was used for data management, statistical analysis, and modeling. All p-values <0.05 or 95% confidence intervals (CI) not including one were considered statistically significant.

## Results

Of the 66,493 stage II and stage III colon cancer patients included in this study, 90.30% had private insurance and 9.70% had Medicaid prior to the PSM. Of the 6,167 pairs of patients included after PSM, 50% had private insurance and 50% had Medicaid. The demographic and clinical characteristics of the patient population prior to and after PSM are summarized in Table 1. Before PSM, excluding stage and distance traveled, there was a statistically significant difference between private insurance and Medicaid patients in terms of gender, race, age, year of diagnosis, education, income, comorbidity, education, facility type, diagnosing/treating facility, treatment delay, and chemotherapy (p<0.0001). For example, before PSM, 22.2% and 30.2% of colon cancer patients under private insurance and Medicaid, respectively, had comorbidities, and this was determined to be a significant factor in colon cancer survival.

However, after PSM with exact match variables on gender, age, race, and stage, there was no statistically significant difference when comparing between private insurance and Medicaid for all other tested variables. For example, after PSM, 30.36% and 30.39% of colon cancer patients under private insurance and Medicaid, respectively, had comorbidities, and these values were not statistically significant (p=0.9844). The same process can be applied to all variables to conclude that the final group of private and Medicaid patients had, among other variables, a similar demographic and socioeconomic status, clinical characteristics, and treatment and that any difference in analyzed survival should now be reliant on payer status.

**Table 1:** Demographics and clinical characteristics of patients with private and Medicaid insurance before and after PSM CCCP: Comprehensive Community Cancer Program; AJCC: American Joint Committee on Cancer; PSM: propensity score matching

Characteristics	Subtypes	Prior to PSM (n=60,042, 6,451)					After PSM (n=6,167, 6,167)				
		Private		Medicaid		P-value	Private		Medicaid		P-value
		N	%	N	%		N	%	N	%	
Gender	Male	30,927	51.51	3,064	47.50	<0.0001	2,924	47.41	2,924	47.41	1.0000
	Female	29,115	48.49	3,387	52.50		3,243	52.59	3,243	52.59	
Race	White	52,420	87.31	4,616	71.55	<0.0001	4,401	71.36	4,401	71.36	1.0000
	Black	7,622	12.69	1,835	28.45		1,766	28.64	1,766	28.64	
Age (years)	40-64	45,657	76.04	5,181	80.31	<0.0001	2,924	47.41	2,924	47.41	1.0000
	≥65	14,385	23.96	1,270	19.69		3,243	52.59	3,243	52.59	
Year of diagnosis	2004-2009	32,691	54.45	2,891	44.81	<0.0001	2,746	44.53	2,750	44.59	0.9567
	2010-2014	27,351	45.55	3,560	55.19		3,421	55.47	3,417	55.41	
Distance traveled (miles)	<30	50,617	85.21	5,443	85.11	0.8385	5,293	85.82	3,064	49.77	0.2302
	≥30	8,788	14.79	952	14.89		874	14.18	3,103	51.34	
Income (USD thousand)	<36	16,310	28.12	3,179	50.48	<0.0001	3,118	50.56	3,111	50.45	0.9140
	≥36	41,685	71.88	3,118	49.52		3,049	49.44	3,056	49.55	
Comorbidity	No	46,708	77.79	4,503	69.80	<0.0001	4,295	69.64	4,293	69.61	0.9844
	Yes	13,334	22.21	1,948	30.20		1,872	30.36	1,874	30.39	
Education	≥20%	21,702	37.42	3,810	60.51	<0.0001	3,753	60.86	3,720	60.32	0.5554
	<20%	36,287	62.58	2,487	39.49		2,414	39.14	2,447	39.68	
Facility type	CCCP	38,289	63.77	3,280	50.84	<0.0001	3,180	51.56	3,140	50.92	0.4823
	Academic/research	21,753	36.23	3,171	49.16		2,987	48.44	3,027	49.08	
Diagnosing/treating facility	Same facility	37,272	62.08	4,313	66.86	<0.0001	4,173	67.67	4,190	67.94	0.7578
	Different facility	22,770	37.92	2,138	33.14		1,994	32.33	1,977	32.06	
Treatment delay (days)	0-30	49,830	84.86	5,193	81.95	<0.0001	5,107	82.81	5,060	82.05	0.2764
	≥31	8,893	15.14	1,144	18.05		1,060	17.19	1,107	17.95	
AJCC stage	II	27,128	45.18	2,898	44.92	0.6929	2,777	45.03	2,777	45.03	1.0000
	III	32,914	54.82	3,553	55.08		3,390	54.97	3,390	54.97	
Chemotherapy	No	21,955	36.57	2,494	38.66	<0.0001	2,349	38.09	2,389	38.74	0.4703
	Yes	38,087	63.43	3,957	61.34		3,818	61.91	3,778	61.26	

**Figure 1:**
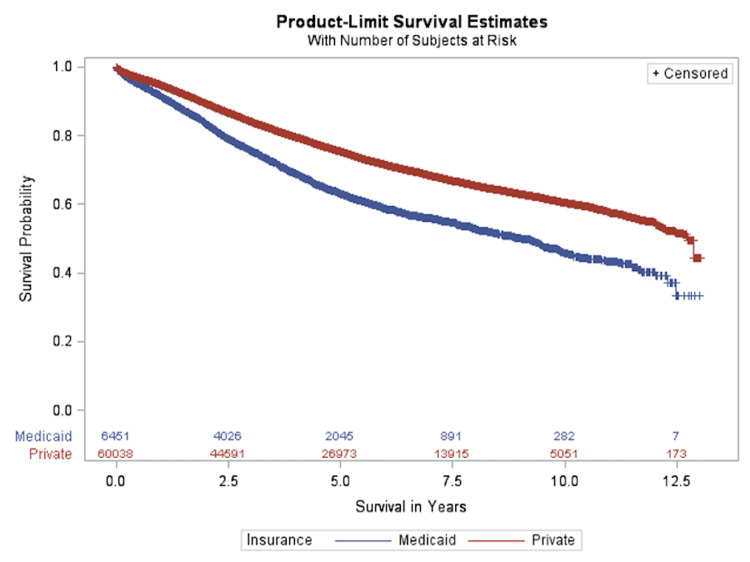
Overall survival according to payer status prior to propensity score matching

**Figure 2:**
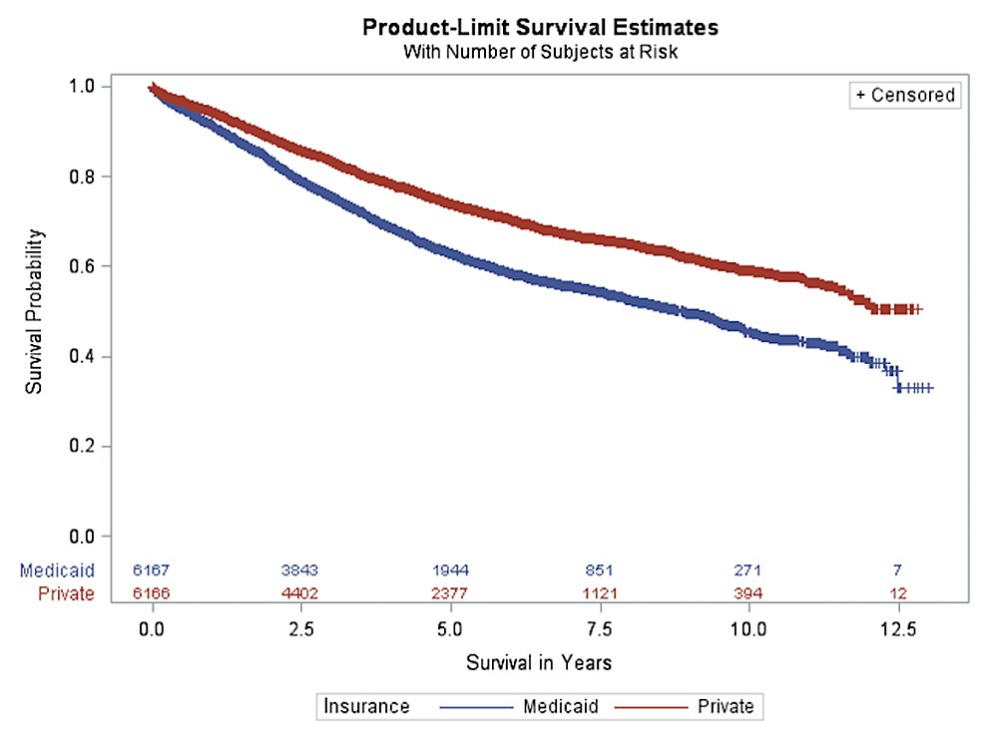
Overall survival according to payer status after propensity score matching

In univariate analysis, OS according to payer status before and after PSM is presented in Figure 1 and Figure 2. Figure 1 shows that prior to PSM, the median overall survival (MOS) was approximately 9.02 years for Medicaid patients, while patients with private insurance had a MOS of 12.75 years.

Figure 2 illustrates the OS by payer status after PSM. The MOS was approximately 8.88 years for patients with Medicaid, while patients with private insurance had a MOS of more than 12.82 years. Thus, even with matching patient characteristics, payer status was found to play a role in the survival of the patients.

Table 2 displays the HR and the 95% CI of HR for each variable from multivariate Cox regression analysis prior to and after PSM. Payer status was a significant predictor of OS (p<0.0001) after controlling for gender, race, age, year of diagnosis, distance traveled, income, comorbidity, education, facility type, diagnosing/treating facility, treatment delay, stage, and chemotherapy before PSM.

In multivariate Cox regression analysis, after adjusting for other factors, the HR prior to PSM for Medicaid patients was 1.54 compared to patients with private insurance. In other words, when compared to private insurance patients, patients with Medicaid were 54% more likely to die. After PSM, the HR was 1.50 for patients with Medicaid compared to patients with private insurance. Similar results were found after adjusting for factors including gender, race, age, year of diagnosis, distance traveled, income, comorbidity, education, facility type, diagnosing/treating facility, treatment delay, stage, and chemotherapy.

After PSM, a majority of factors including race, year of diagnosis, distance traveled, income, education, facility type, diagnosing/treating facility, and treatment delay were found to be significant predictors of OS.

**Table 2:** Multivariate analysis of predictors of overall survival prior to and after PSM CCCP: Comprehensive Community Cancer Program; AJCC: American Joint Committee on Cancer; PSM: propensity score matching; HR: hazard ratio; CI: confidence interval

Characteristics	Subtypes	Prior to PSM				After PSM			
		HR	95% CI		P-value	HR	95% CI		P-value
			Lower	Upper			Lower	Upper	
Insurance	Private	1.00							
	Medicaid	1.54	1.47	1.62	<0.0001	1.50	1.41	1.60	<0.0001
Gender	Female	1.00							
	Male	1.17	1.14	1.21	<0.0001	1.22	1.14	1.30	<0.0001
Race	White	1.00							
	Black	1.14	1.09	1.19	<0.0001	1.08	1.01	1.16	0.0354
Age (years)	40-64	1.00							
	≥65	2.24	2.17	2.32	<0.0001	1.70	1.58	1.83	<0.0001
Year of diagnosis	2010-2014	1.00							
	2004-2009	1.06	1.03	1.10	0.0005	1.12	1.05	1.21	0.0014
Distance traveled (miles)	<30	1.00							
	≥30	1.08	1.03	1.13	0.001	1.11	1.02	1.22	0.0236
Income (USD thousand)	≥36	1.00							
	<36	1.10	1.05	1.14	0	1.13	1.05	1.22	0.0017
Comorbidity	No	1.00							
	Yes	1.43	1.39	1.48	<0.0001	1.38	1.29	1.47	<0.0001
Education	≥20%	1.00							
	<20%	0.99	0.96	1.03	0.7354	1.01	0.93	1.09	0.8461
Facility type	Academic/research	1.00							
	CCCP	1.06	1.03	1.10	0.0001	1.08	1.01	1.15	0.0204
Diagnosing/treating facility	Different facility	1.00							
	Same facility	1.19	1.15	1.24	<0.0001	1.10	1.02	1.18	0.0186
Treatment delay (days)	≥31	1.00							
	0-30	1.10	1.05	1.15	<0.0001	1.18	1.08	1.30	0.0003
AJCC stage	II	1.00							
	III	2.29	2.20	2.38	<0.0001	2.15	1.98	2.33	<0.0001
Chemotherapy	Yes	1.00							
	No	1.83	1.76	1.90	<0.0001	1.91	1.76	2.07	<0.0001

## Discussion

This study effectively demonstrates that payer status has a statistically significant effect on the OS of colon cancer patients after adjusting for all other predictive factors through the PSM method. Medicaid patients had a 50% increased risk of death compared to those with private insurance. Our findings are consistent with previous studies on payer status and cancer patient survival [1,3,5-7,36]. Although the mechanism by which payer status affects OS is not entirely clear, it could be mediated, among numerous other variables, through differences in access to certain treatment types [40], proper screenings [41], or improper follow-up following a diagnosis [1]. Further research may be warranted to investigate this observation through mediation analysis.

Similar to findings from other studies, we observed that patients with higher incomes and higher education had better survival from cancer [30,42,43]. In addition, our data agree with other studies that show that male gender, advanced age, African American race, and a higher Charlson Comorbidity score are associated with decreased OS [18,27,44,45]. Our findings are also in line with the results of previous studies regarding the effect of tumor stage and treatment [43,46], distance traveled [30], and year of diagnosis [18] on patient survival. Finally, other factors (facility type, treatment delay, and diagnosing/treating facility) also showed an association with OS, consistent with findings in the current literature [47]. All the above predictive factors were adjusted for in our final analysis while assessing the effect of payer status on patient OS. Overall, we determined that payer status has a statistically significant relationship with OS.

Prior to PSM, it was discovered that patient demographics, socioeconomic status, and clinical characteristic were significantly different between private and Medicaid patient groups (Table 1). Differences in survival between private insurance patients and Medicaid patients were already found before PSM (Figure 1). Thus, the difference found in survival may not be completely due to payer status but could also be due to differences in other factors such as gender, race, age, and comorbidities. For example, prior to PSM, 22.2% and 30.2% of colon cancer patients under private insurance and Medicaid, respectively, had comorbidities. Other studies have indicated that the presence of more comorbidities leads to a shorter survival [39]. All other factors except distance traveled and stage have been shown to have a significant effect on colon cancer survival including male gender [18], black race [19,22], and old age [18], which have all been linked to a worse survival rate.

After PSM, the differences in patient characteristics and clinical status listed above were not found to be statistically significant between private insurance and Medicaid. For example, after PSM, 30.36% and 30.39% of patients under private insurance and Medicaid, respectively, had comorbidities (p=0.9844). Other factors like gender, race, age, and stage were exact matches and had p-values of 1.000. Therefore, it was determined that the difference in survival between private insurance and Medicaid colon cancer patients (Figure 2) is due to the difference in payer status alone.

In multivariate Cox regression analysis with PSM, it was shown that patients with Medicaid were 50% more likely to die compared to private insurance patients. In addition, we demonstrated that patients who do not receive chemotherapy are 91% more likely to die compared to patients who receive chemotherapy. After PSM in multivariate Cox regression analysis, covariates such as gender, race, age, year of diagnosis, distance traveled, income, comorbidity, education, facility type, diagnosing/treating facility, treatment delay, and stage were controlled for and payer status was found to be a significant factor in the survival of colon cancer patients.

Despite the efforts to account for as many confounding variables as possible while utilizing a large sample population, there are still some limitations to this study. Due to the limited number of variables that we could apply in our analysis, there may still be a few important confounding variables that we could not control for. Furthermore, this study only investigated stage II and stage III colon cancer patients who had undergone surgery and chemotherapy as treatment. Hence, the results from this study may not apply to other patient populations. Data on Education and income were collected by ZIP Code rather than by patient or household. Utilizing individual or household income in the analysis would have strengthened the results. Information regarding specific causes of death was also not available in the NCDB file. Measuring the effect of payer status on cause-specific survival might yield different results.

## Conclusions

This study has indicated that after PSM, patient demographics, socioeconomic status, and clinical characteristics were similar between private insurance and Medicaid patients. However, the survival differences between these two groups still remained after PSM, and it was determined that it could be caused by the effect of the payer status. Payer status was found to play a role in the survival of colon cancer patients even after adjusting for other variables. These timely findings shed light on the significant impact of payer status on the overall survival of colon cancer patients. As we continue to navigate our way through the dynamically changing landscape of healthcare reform in the United States today, it is important to consider the influence of payer status on health outcomes in the future.
